# Distinguishing highly similar gene isoforms with a clustering-based bioinformatics analysis of PacBio single-molecule long reads

**DOI:** 10.1186/s13040-016-0090-8

**Published:** 2016-04-05

**Authors:** Ma Liang, Castle Raley, Xin Zheng, Geetha Kutty, Emile Gogineni, Brad T. Sherman, Qiang Sun, Xiongfong Chen, Thomas Skelly, Kristine Jones, Robert Stephens, Bin Zhou, William Lau, Calvin Johnson, Tomozumi Imamichi, Minkang Jiang, Robin Dewar, Richard A. Lempicki, Bao Tran, Joseph A. Kovacs, Da Wei Huang

**Affiliations:** Critical Care Medicine Department, Clinical Center, Frederick, MD USA; Leidos BioMedical Research, Inc., Frederick National Laboratory for Cancer Research, NIH, Frederick, MD USA; Center of Information Technology, National Institutes of Health (NIH), Bethesda, MD USA; Current Affiliation: National Cancer Institute, NIH, Bethesda, MD USA

**Keywords:** PacBio, Bioinformatics analysis, Gene isoforms, Repetitive sequences, Major surface glycoprotein, *Pneumocystis*, NGS, Uclust

## Abstract

**Background:**

Gene isoforms are commonly found in both prokaryotes and eukaryotes. Since each isoform may perform a specific function in response to changing environmental conditions, studying the dynamics of gene isoforms is important in understanding biological processes and disease conditions. However, genome-wide identification of gene isoforms is technically challenging due to the high degree of sequence identity among isoforms. Traditional targeted sequencing approach, involving Sanger sequencing of plasmid-cloned PCR products, has low throughput and is very tedious and time-consuming. Next-generation sequencing technologies such as Illumina and 454 achieve high throughput but their short read lengths are a critical barrier to accurate assembly of highly similar gene isoforms, and may result in ambiguities and false joining during sequence assembly. More recently, the third generation sequencer represented by the PacBio platform offers sufficient throughput and long reads covering the full length of typical genes, thus providing a potential to reliably profile gene isoforms. However, the PacBio long reads are error-prone and cannot be effectively analyzed by traditional assembly programs.

**Results:**

We present a clustering-based analysis pipeline integrated with PacBio sequencing data for profiling highly similar gene isoforms. This approach was first evaluated in comparison to *de novo* assembly of 454 reads using a benchmark admixture containing 10 known, cloned *msg* genes encoding the major surface glycoprotein of *Pneumocystis jirovecii.* All 10 *msg* isoforms were successfully reconstructed with the expected length (~1.5 kb) and correct sequence by the new approach, while 454 reads could not be correctly assembled using various assembly programs. When using an additional benchmark admixture containing 22 known *P. jirovecii msg* isoforms, this approach accurately reconstructed all but 4 these isoforms in their full-length (~3 kb); these 4 isoforms were present in low concentrations in the admixture. Finally, when applied to the original clinical sample from which the 22 known *msg* isoforms were cloned, this approach successfully identified not only all known isoforms accurately (~3 kb each) but also 48 novel isoforms.

**Conclusions:**

PacBio sequencing integrated with the clustering-based analysis pipeline achieves high-throughput and high-resolution discrimination of highly similar sequences, and can serve as a new approach for genome-wide characterization of gene isoforms and other highly repetitive sequences.

**Electronic supplementary material:**

The online version of this article (doi:10.1186/s13040-016-0090-8) contains supplementary material, which is available to authorized users.

## Background

Gene isoforms are commonly found in both prokaryotes and eukaryotes organisms, and can be produced from different but closely related genes (known as multi-copy gene families) or from the same gene by alternative splicing. For example, alternative splicing of the human genome can result in greater than 100,000 unique isoforms from only ~20,000 genes [1–4]. Particularly, the single titin gene in humans as well as other mammals can generate millions of alternatively spliced isoforms [2, 5]. The production of these isoforms can increase the structural and functional diversity of gene products, and result in profound effects in cellular processes, developmental states, tissue or cell specificity, and disease conditions [1–5]. In contrast to mammals, low complex organisms, especially microorganisms, have significantly lower rates of alternative splicing but are rich in multi-copy gene families. The most prominent examples include the variant surface glycoprotein (*vsg*) gene family (~1,000 members) in *Trypanosoma brucei* [6], the variant surface protein (*vsp*) gene family (~235–275 members) in *Giardia lamblia* [7], the *var* gene family (~60 members) in *Plasmodium falciparum* [8], and the major surface glycoprotein (*msg*) gene family (estimated ~80 members) in *Pneumocystis* [9–11]. All these gene families encode variable surface protein isoforms, which are thought to be employed by the microorganisms to generate antigenic variation in order to evade the host immune response and, in some cases, to maintain persistent infections [12].

**Fig 1:**
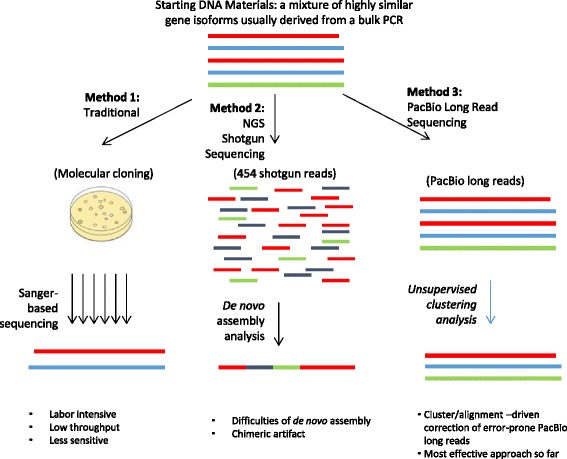
Comparison of three methods for gene isoform identification. Lines with different colors represent different sequences

While studying the dynamics of gene isoforms is important in understanding biological processes and disease conditions, characterization of the full complement of isoforms within targeted genes or across an entire genome is technically challenging due to the high degree of sequence identity among these isoforms. Traditionally, gene isoforms have been identified primarily by Sanger sequencing of cloned DNA or cDNA fragments (e.g. by PCR or hybridization selection. Fig. 1: Method 2). This approach is very tedious and time-consuming, and has low throughput, thus not suitable for large-scale screening of isoforms across various biological processes or disease conditions. In addition, this approach is not sensitive to low abundant isoforms. Due to these limitations, characterization of gene isoforms in many organisms is limited.

Next generation sequencing (NGS) technologies, such as Roche 454 and Illumina platforms, significantly increase the efficiency of sequencing by producing a large number of 100–600 bp reads rapidly and with a high throughput [13]. While these NGS methods are very successful when applied to the sequencing of genomes, exomes and transcriptomes as well as to single nucleotide polymorphism (SNP) discovery [13, 14], accurate characterization of gene isoforms with highly similar sequences remains problematic. The major bottleneck is that NGS read lengths are usually substantially shorter than the length of typical gene isoforms, and thus require assembly, which may result in inaccurate joining of highly similar short reads from different isoforms to form artificial chimeric sequences (exemplified in Fig. 1: Method 2). Most of the currently available assembly software packages (such as 454’s Newbler, Celera Assembler, CLC Bio, MIRA, MINIMUS, Vicuna, Trinity, PredictHaplo, and Cufflinks) [15, 16], were developed for general assembly purposes and have proven to be successful in many NGS studies of diploid/haploid genome assemblies, but their performance is poor in the assembly of highly similar gene isoforms or other repetitive sequences [16–19].

The history of *Pneumocystis msg* isoform discovery in the past decade reflects the aforementioned challenges for gene isoform identification. We have long been interested in studying the *msg* genes in *P. jirovecii*, an opportunistic pathogen of immunosuppressed patients [20, 21]. Using traditional Sanger sequencing of cloned DNA fragments, we identified a limited number of full-length *msg* gene isoforms in *P. jirovecii* [22, 23]. Each *msg* gene is ~3 kb in length. The overall average identity among *msg* isoforms is 75 %, with a range from 57 % to 91 % [22]. While a limited number of *msg* genes has been also identified in all other *Pneumocystis* species examined (reviewed by Stringer [11]), the exact number and sequence diversity of the *msg* members in any species remains unknown due largely to the lack of an efficient sequencing method. Even in the current genome assemblies of both *P. carinii* [24] and *P. jirovecii* [25], which were sequenced by traditional Sanger method and NGS technologies, respectively, the *msg* genes have been almost entirely excluded due to failure in assembly of highly similar short reads for msg genes. The lack of complete sequences of the *msg* family has hampered our understanding of its diversity, recombination mechanisms and biological functions.

**Fig 2:**
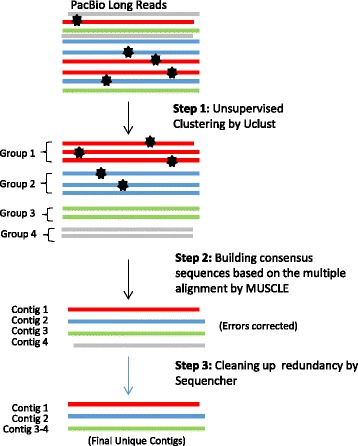
Schema of the clustering-based data analysis procedure using PacBio long reads. The different colors represent the reads belonging to different isoforms. The black stars represent sequencing errors. In this example, contig 3 and 4 are merged because they are the plus and minus strands of the same isoform

The newly emerging PacBio sequencing platform, a third-generation sequencer (TGS), is capable of sequencing tens of thousands of individual DNA molecules in parallel and in real time [26, 27]. The most distinctive feature of PacBio is the generation of very long reads (up to ~40 kb), which cover the full length of typical genes and gene families, thus minimizing or eliminating the need for sequence assembly. However, the PacBio long reads are error-prone [28] and cannot be effectively analyzed by standard sequence alignment and assembly programs [27, 29]. In this paper, we describe a unique clustering-based computational pipeline for analyzing the long but error-prone PacBio reads, and demonstrate its capacity to achieve high-throughput and high-resolution profiling of the *msg* isoforms of *P. jirovecii* (Fig. 1: Method 3). The key to this approach is that the PacBio long reads enable each targeted isoform (in a mixture of all targeted isoforms amplified by PCR) to be continuously sequenced multiple times at its full length or nearly full length, which eliminates the challenging *de novo* assembly process required for highly similar short reads generated by other NGS methods. The full-length sequence for each isoform is obtained by an unsupervised clustering-based analysis pipeline during which the randomly distributed PacBio read errors can be largely corrected as demonstrated in the paper (Fig. 2).

## Methods

### Two benchmark isoform-admixture samples containing known, cloned *P. jirovecii msg* genes

**Fig 3:**
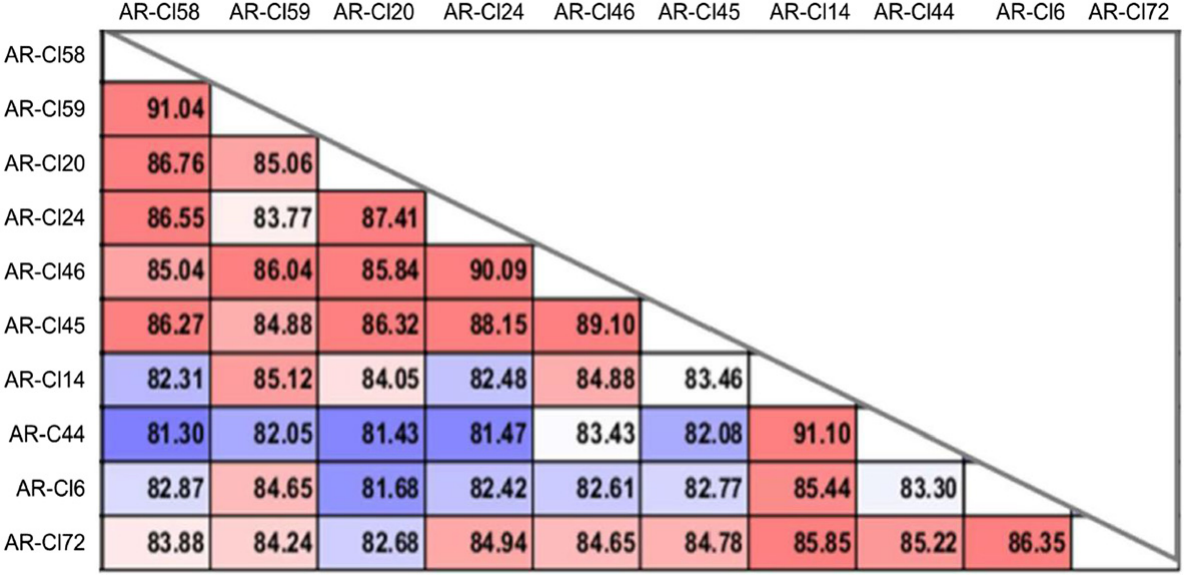
High sequence identity of 10 *msg* isoforms of *P. jirovecii* previously identified by Sanger sequencing of plasmid-cloned PCR products [22]. Text labels on the left side and the top represent plasmid ID. Colors indicate different degrees of identity from low (blue) to high (red). Plasmids containing these 10 isoforms were mixed together to form a benchmark admixture, which was amplified by PCR and sequenced in parallel by PacBio and 454 sequencing

Ten plasmids, representing different *msg* gene isoforms (~3 kb each) previously identified from a *P. jirovecii* strain by traditional Sanger sequencing of cloned PCR amplicons [22, 29], were mixed together to form an artificial admixture of highly similar isoforms with 81–91 % identity (Fig. 3). This admixture served as a benchmark sample mimicking a gene family in vivo. Degenerate PCR primers were then used to amplify an ~1.5 kb portion of the *msg* isoforms in a bulk PCR reaction as described in an earlier paper [22]. The bulk PCR product was sequenced in parallel by the Roche 454 and PacBio platforms. The ~1.5 kb fragment was chosen as it was the optimal size for PacBio sequencing at the time our work began (see Additional file 3 fasta sequence file). As the PacBio system improved, we tested its ability to sequence the full-length *msg* isoforms (~3 kb) using another benchmark admixture containing 22 of the 24 plasmid clones representing unique *msg* gene isoforms previously cloned from a different *P. jirovecii* strain [22]. This plasmid admixture was amplified by PCR targeting the full-length *msg* genes (~3 kb each) using a pair of degenerate primers JSG.f1, 5’-TGGCGCGGGCGGTYAAGCGG-3’, and JSG.r1, 5’-YRCCCCYCTCATCAC-3’, and conditions described elsewhere [22].

### Genomic DNA from a clinical strain of *P. jirovecii*

*Human subject experimentation guidelines of the National Institutes of Health were followed in the conduct of these studies. P. jirovecii* genomic DNA was extracted from the same patient lung sample used in a previous study to clone 24 *msg* isoforms [22], which were used to make the second benchmark admixture as described above. This DNA sample was used to amplify the full-length coding regions of *msg* genes. To maximize the coverage of *msg* genes, two pairs of primers were designed from highly conserved regions at the 5’ and 3’ ends of ~30 known *P. jirovecii msg* genes [22, 23], including the primer pair JSG.f1 and JSG.r1 described above and another pair GK126a (5’-GGCGCGGGCGGTGGCGCGGGCGGT-3’) and GK452 [22]. These two pairs of primers were used in separate PCR reactions following conditions described elsewhere [22]. Equal amount of PCR products were pooled from each reaction and sequenced by the PacBio sequencing platform as described below.

### Roche 454 and PacBio sequencing

For 454 sequencing, the input DNA samples (PCR amplicons) were fragmented using a Covaris sonicator before library construction. A standard shotgun procedure was used for all steps of 454 library construction and deep sequencing according to the manufacturer’s standard shotgun protocols. For PacBio sequencing, the input DNA samples (PCR amplicons) were used directly (without fragmenting) for circular consensus sequences (CCS) library construction (with a 1.5 kb CCS library for the ~1.5 kb amplicon and a 3 kb CCS library for the ~3 kb amplicon). All CCS libraries were sequenced following the manufacturer’s standard protocols, using either XL or P4 polymerase chemistry, and 90 minute movies.

### Bioinformatics analysis of 454 reads

Raw 454 sff files were converted to fastq files using the standard 454 software package. The 454 reads were filtered with QC criteria of read length ≥ 50 bp, average quality score ≥ 30, and total Ns ≤ 3. The key bioinformatics analysis was *de novo* assembly of the short reads (50–600 bp) into the full length sequences (~1.5 or ~3 kb) of the input amplicons (Fig. 1: Method 2). In order to obtain the best possible results, the qualified reads were subjected to *de novo* assembly using various software packages including 454’s Newbler, Celera Assembler, CLC Bio, MIRA, MINIMUS, Trinity, Vicuna, PredictHaplo, and Cufflinks [15]. These assembly programs cover the popular assembly algorithms including the *de Bruijn* graph algorithm and overlap-to-consensus [15]. We first tested these packages with the default parameters, and then adjusted the parameters from lower to higher stringencies. The resulting contigs from various programs and parameters were compared to the known *msg* sequences in the benchmark isoform-admixture sample.

### A unique clustering-based analysis pipeline for PacBio long reads

PacBio raw movie files were converted into CCS reads in fastq format using the standard PacBio SMRT software package. The reads were filtered with QC criteria of read length ≥ 1 kb and < 3.5 kb. Given that the PacBio reads should represent the full length or nearly full length of the input amplicons, the key process was to classify these reads rather than assemble them as required with the 454 reads. The unique clustering-based analysis pipeline consisted of the following steps (Fig. 2 and Additional file 2: Table S2 for details):

Firstly, all raw CCS reads were clustered into different groups of reads based on their global similarities using the Uclust [30] program (similarity threshold set to 0.9). Uclust is a sequence clustering program mainly used in phylogenetic analysis to congregate similar sequences into groups [30] and has not been used previously for NGS data analysis. We are the first group to adopt it for PacBio sequencing analysis and found Uclust’s speed to be fast enough to sort ~10,000 sequences in several minutes. It is theoretically able to output any number of clusters as driven by the input dataset itself. Of note, the Uclust procedure is completely unsupervised, and relies on classifying full length or near full length reads so that it only concentrates on the unsupervised clustering process without any extra effort to build internal linkages between reads. Thus, this procedure is more suitable for PacBio long reads than for NGS short reads.

Secondly, the input of groups of reads was summarized into each corresponding contig. The reads in each group clustered from the first step were aligned using the MUSCLE [31] program (with default parameters), which resulted in a consensus sequence or contig for each group. At this point, the first and second steps not only reduced redundancy by merging tens of thousands of PacBio reads into a relatively small number of contigs, but also corrected the sequencing errors in individual PacBio reads. The error correction power relied on the procedure of building consensus sequences from multiple alignments since the errors in each individual read were randomly distributed.

Finally, the redundancies of contigs above in either plus or minus strand were further merged into unique contigs. We used the program Sequencher (Gene Codes Corporation, Ann Arbor, MI) (Similarity = 95 % and overlap = 100 bp) to perform the final merging/cleanup step in order to remove redundancies such as plus and minus strands of the same isoform, as well as to filter out singleton contigs. At this point, each contig in the output represented a unique gene isoform. All isoforms identified were supported by sequences from both directions.

### Evaluation of contigs

To examine the accuracy of the final contigs corresponding to the known *msg* isoforms, the NCBI BLAST program was used to match the final contig sequences derived from the various analyses described above with all known *P. jirovecii msg* genes. Identity (0–100 %) and length (relative to known *msg* genes) were used to determine the level of accuracy of contigs. To examine the accuracy of contigs containing new *msg* isoforms identified from the clinical sample, six novel contigs were selected for verification by PCR using isoform-specific primers and original genomic DNA; PCR products were sequenced by Sanger sequencing. All *msg* isoforms identified in this study have been incorporated into the *P. jirovecii* RU genome assembly (GenBank under accession no. PRJNA223510) [32].

## Results and discussion

### Study design

Figure 1 illustrates three approaches for gene isoform characterization, including the traditional Sanger sequencing of plasmid-cloned PCR products (Method 1), 454 sequencing followed by *de novo* assembly (Method 2) and PacBio sequencing followed by clustering analysis (Method 3). We have previously reported the use of the Method 1 to identify a limited number of *P. jirovecii msg* isoforms [22]. In the present study, we compared the performance of the latter two methods using two benchmark isoform admixtures containing 10 or 22 previously cloned *P. jirovecii msg* isoforms. The performance of each method was evaluated by measuring the number, length and sequence identity of *msg* isoforms reconstructed (relative to the known isoforms in the benchmark isoform admixtures used). The performance of Method 3 was further assessed by applying it to the original clinical sample from which the 22 known *msg* isoforms were identified previously by the Method 1, with an expectation of identifying all known isoforms as well as additional new isoforms.

### Evaluation of method 2 – Roche 454 sequencing followed by *de novo* assembly using the benchmark sample

The ~1.5 kb amplicon from the benchmark admixture containing 10 known *msg* isoforms was sequenced using the 454 shotgun protocol, yielding ~47,000 pass-filter reads with an average length of 350 bp (ranging from 50 to 400 bp). By aligning to the 10 known sequences, we found that these reads fully covered the ~1.5 kb amplified region for each of the 10 known isoforms in the admixture. We attempted *de novo* assembly of these reads using various *de novo* assemblers, including 454’s Newbler, Celera Assembler, CLC Bio, MIRA, MINIMUS, Vicuna, Trinity, and Cufflinks [15, 18]. As shown in Additional file 1: Table S1, the contigs generated by these assemblers shared the following issues: 1) Only a few (1–2) contigs contained the expected ~1.5 kb sequences though a total of 10 contigs were expected. 2) Most of the remaining contigs were substantially shorter than ~1.5 kb, and showed < 90 % identity to any of the known sequences. Detailed examination of these contigs revealed false joinings, that is, artificial chimeric sequences formed by reads from different isoforms during *de novo* assembly. Chimeric contigs are a common problem encountered in *de novo* assembly of highly similar isoforms [17, 19]. Despite adjusting the parameters of the assembly programs in many different ways from lower to higher stringencies, the improvements were limited.

Our experience above is consistent with previous studies showing that the *de no*vo assembly of short, highly similar reads from NGS platforms for gene isoforms is a very difficult task [17, 19]. The challenges come from several aspects [15]: 1) Most of the assembly programs were designed to tolerate a certain degree of read ‘error’, i.e. treating slightly different reads as the same sequence. However, in isoform assembly, such error toleration is problematic as it overlooks true biological variants and may result in compressing slightly different reads from different isoforms into one artificial chimeric contig. Such chimeric contigs cannot be easily eliminated by increasing the stringency through changing the parameters of the assembly programs, due to the difficulty in drawing an appropriate line for the signal-to-noise ratio. Indeed, the line is dynamic from read to read, i.e. reads may have more or less variants depending on the variant distribution along the regions for the given isoforms. 2) Most of the assembly programs were built with the assumption that the sequences are coming from diploid or haploid systems. Thus, they tend to assemble sequence reads into long contigs/scaffolds based on a majority rule without considering the alternatives despite clearly different patterns in minorities in some cases. For example, in order to assemble many reads derived from a bacterial colony, the assemblers tend to generate a single contig while ignoring unmatched reads, which is reasonable under the assumption that one bacteria colony should have only one genome. Thus, such assemblers are generally not able to assemble reads that are related to but different from the majority reads. 3) Some assembly programs (such as Trinity, Vicuna, Cufflinks or PredictHaplo) [19] do indeed consider the possibility of multiple isoforms during *de novo* assembly and have the ability to produce multiple contigs [33]. However, in our experience and that of others [18], these programs are still unreliable for highly similar isoform characterization. The performance of these assemblers largely depends on factors such as the distribution pattern of sequencing errors (scattered everywhere *vs*. concentrated in small areas), the proportion of each isoform in the pool, and the level of sequence identity among the isoforms [18].

Due to the failure to reconstruct the ~1.5 kb segment of the 10 known *msg* isoforms using 454 reads from the benchmark admixture, we did not repeat this experiment using the other benchmark admixture containing 22 known *msg* isoforms to reconstruct their ~3 kb full-length, which would likely have been even less successful. Our failure to reconstruct the known *msg* isoforms using the benchmark admixture mirrored the inability of other investigators to identify *msg* isoforms by assembly of whole genome sequence reads generated by 454 and Illumina platforms for *P. jirovecii* [25] and by Sanger sequencing for *P. carinii* [10, 24].

### Evaluation of method 3 -PacBio sequencing integrated with a unique clustering-based analysis pipeline using two benchmark samples and one clinical sample

The same ~1.5 kb amplicon used in the Method 2 described above was sequenced using the PacBio platform, yielding ~8,500 pass-filter CCS reads with the length in the range of 0.5 to 1.5 kb. We attempted *de novo* assembly of these CCS reads using the same set of assembly programs applied in Method 2. Surprisingly, none of these programs was able to reconstruct any one of the 10 expected *msg* isoforms, with all contigs containing numerous ambiguous sequences resulting from higher errors in individual CCS reads or false joining of CCS reads from different isoforms.

**Fig 4:**
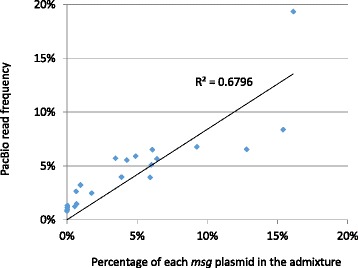
The sensitivity of the new approach based on PacBio sequencing and clustering analysis in detection of minor gene isoforms in a mixture containing multiple isoforms. Twenty-two plasmids, representing 22 different *P. jirovecii msg* isoforms previously cloned [22], were mixed with various concentrations and amplified by PCR for the full-length *msg* coding region (~3 kb) followed by PacBio sequencing and clustering analysis. The read frequency and concentrations of plasmids (indicated by diamonds) in the mixture are positively correlated. The concentration is the percentage of each plasmid DNA relative to the total amount of plasmid DNA in the mixture. Those 4 *msg* isoforms, which were not identified, have the lowest concentrations (0.8–1.3 %) in the mixture

To take advantage of the PacBio long reads and to tolerate their high error rate, we constructed a unique clustering-based analytical pipeline as described in the Methods and illustrated in Fig. 2. When this pipeline was applied to the ~1.5 kb PacBio CCS reads, it generated 10 contigs whose sequences had a one-to-one match with the 10 expected *msg* isoforms in the benchmark sample at an identity level greater than 99.5 % (Table 1). When it was further applied to the ~3 kb PacBio CCS reads from the benchmark admixture containing 22 of the 24 known *msg* isoforms, it reconstructed all but 4 of the 22 isoforms in their full length with an identity > 99.4 %; the 4 isoforms that were not reconstructed were present at concentrations ≤1.3 % in the admixture. This finding suggests that this approach has a detection threshold of ~1 % (Fig. 4).

**Table 1:** Reconstruction of an ~1.5 kb segment of 10 known *msg* isoforms of *P. jirovecii* from the benchmark isoform admixture by PacBio sequencing and clustering-based analysis (see Additional file 2: Table S2 and Additional file 3 for more details)

Contig no.	Length^a^ (bp)	Matched msg isoform	Identity (%)
Contig0001	1587	AR-Cl72	100
Contig0002	1581	AR-Cl59	99.94
Contig0003	1584	AR-Cl46	99.94
Contig0004	1584	AR-Cl14	99.87
Contig0005	1581	AR-Cl6	99.75
Contig0006	1581	AR-Cl24	99.87
Contig0007	1593	AR-Cl20	99.94
Contig0008	1584	AR-Cl44	99.81
Contig0011	1584	AR-Cl45	99.94
Contig0014	1583	AR-Cl58	99.87

^a^Identical between the contigs and known *msg* isoforms

The success of the new approach with the two benchmark samples prompted us to directly apply it to the original clinical sample from which the 24 known *msg* isoforms of *P. jirovecii* were identified previously [22] by Method 1 as described above. After PCR amplification of the *msg* repertoire in its full-length (~3 kb) using genomic DNA from this sample, the amplicon was sequenced with the 3 kb PacBio CCS sequencing protocol, yielding ~9,000 pass-filter CCS reads with an length of 1 – 3 kb. These reads were processed by the clustering-based analysis pipeline, resulting in 72 unique contigs, which represent 72 *msg* isoforms (Table 2; Additional file 3: Sequence Data). All contigs contained the full-length coding regions of the *msg* genes. Twenty-four of them exhibited one-to-one matches to the 24 known *msg* isoforms with an identity > 99.4 % (Table 2), supporting the high accuracy and reproducibility of the new approach. The remaining 48 contigs showed high-level sequence identity but were clearly distinct compared to the 24 known *msg* isoforms, and thus they represented novel *msg* isoforms. Six new isoforms were chosen for verification by PCR; all of them were successfully amplified from the original *P. jirovecii* genomic DNA using isoform-specific primers, and the resulting sequences were consistent with the corresponding PacBio contigs, with > 98 % sequence identity (Table 2). The total number of *msg* isoforms (72) identified from this *P. jirovecii* strain is within the estimated 70–80 isoforms based on studies of *P. carinii* [9, 10]. This represents the first report of the complete or near complete set of full-length *msg* isoforms in any *Pneumocystis* species. Our findings support the capacity of the new approach to achieve high-throughput and high-resolution profiling of the *P. jirovecii msg* isoforms in clinical samples.

**Table 2:** A partial list of *P. jirovecii* full-length *msg* isoforms (~3 kb) identified in a clinical sample by PacBio sequencing and clustering-based analysis

Contig no.	Length (bp)	Matched *msg* gene	Status	Identity to the known (%)	PCR verification
Contig0020	3086	EF371022	Known	98.92	ND
Contig0007	3086	EF371023	Known	99.87	ND
Contig0021	3008	EF371024	Known	99.83	ND
Contig0025	3011	EF371025	Known	99.60	ND
Contig0133	3068	EF371026	Known	99.71	ND
Contig0022	3104	EF371028	Known	99.58	ND
Contig0006	3062	EF371029	Known	99.97	ND
Contig0008	3041	EF371030	Known	99.87	ND
Contig0026	3032	EF371031	Known	98.76	ND
Contig0012	3005	EF371032	Known	99.70	ND
Contig0013	2996	EF371033	Known	99.87	ND
Contig0011	3092	EF371035	Known	99.88	ND
Contig0003	3038	EF371036	Known	99.97	ND
Contig0005	3002	EF371038	Known	99.90	ND
Contig0010	2996	EF371040	Known	99.80	ND
Contig0015	3129	EF371041	Known	99.78	ND
Contig0001	3002	EF371042	Known	99.37	ND
Contig0027	3065	EF371045	Known	99.64	ND
Contig0014	3077	EF371050	Known	99.71	ND
Contig0018	3044	EF371051	Known	99.70	ND
Contig0009	3029	EF371052	Known	99.87	ND
Contig0017	3023	EF371053	Known	99.50	ND
Contig0016	3050	EF371055	Known	99.84	ND
Contig0004	3026	EF371056	Known	99.97	ND
Contig0010b	3060	No	Novel	NA	99.53
Contig0004b	3086	No	Novel	NA	99.45
Contig0015b	3039	No	Novel	NA	99.20
Contig0053b	3077	No	Novel	NA	98.91
Contig0006b	3062	No	Novel	NA	98.50
Contig0054b	3074	No	Novel	NA	98.32
Contig0138b	3041	No	Novel	NA	ND

A total of 72 unique *msg* isoforms identified in this study, with only 31 of them shown in this table. The first 24 contigs matched in full-length with the 24 previously identified *msg* genes from the same clinical sample [22] as shown in the third column with GenBank accession no, *NA* not applicable, *ND* not determined by PCR. Additional file 4 contains a complete list of sequences for 72 *Msg* isoforms

In this study, we choose the *P. jirovecii msg* gene family as the model isoform system since it represents the largest surface protein gene family identified to date in non-protozoan microorganisms [12]. Based on restricted fragment length polymorphism analysis [22, 34], the *msg* repertoire is different among different *P. jirovecii* strains. Characterizing the *msg* repertoire in different *P. jirovecii* strains will allow us to examine the phylogenetic relationship among these strains, and to determine if there are associations related to pathogenicity or geography. Given the high sequence identity and the large family size and gene length (~3 kb each) of the *msg* genes, successful characterization of this gene family implies that our approach could be easily adapted to characterize gene isoforms and other highly repetitive sequences in other organisms. Due to the relatively low outputs per run of the PacBio system, a requirement for this approach at present is that the gene families/isoforms must contain highly conserved sequences so that they can be enriched by PCR, hybrid capture [35], or other methods before PacBio sequencing.

## Conclusions

PacBio long reads have obvious advantages over the short reads generated by 454 and other NGS platforms for sequencing highly similar gene isoforms because the long reads cover the full-length of typical genes, allowing elimination of the *de novo* assembly challenges encountered by the NGS short reads. However, such advantages can only be fully exploited for gene isoform characterization when appropriate bioinformatics analyses are applied to distinguish the minor differences and correct the high error rates in PacBio reads. In this study we integrated a clustering-based bioinformatics analysis pipeline with PacBio sequencing, and demonstrated that this approach can efficiently and accurately distinguish very long and highly similarly gene isoforms using the multi-copy *msg* gene family of *P. jirovecii* as a model system. This approach could be easily adapted to characterize gene isoforms and other highly repetitive sequences in other organisms. Indeed, for example, we have successfully adapted this approach to characterize *msg* and kexin gene families in rodent *Pneumocystis* [32].

## Additional files

Additional file 1: Table S1. Representative results of failure of *de novo* assembly of 454 reads for an ~1.5 kb segment of 10 known *msg* isoforms of *P. jirovecii*. (DOCX 18 kb) Additional file 2: Table S2. Explanation of the Uclust-based analysis pipeline in details. (DOC 27 kb) Additional file 3: Sequence Data. Benchmark dataset 1 consisting of raw PacBio reads of 10 known MSG plasmid and the final analysis result. (ZIP 14 kb) Additional file 4: Sequence Data. 72 *P. jirovecii msg* sequences in fasta format. It contains 24 known MSGs and 48 novel MSGs identified by the described analysis pipeline. (FASTA 218 kb)

## Abbreviations

454: 454 Titanium RS
PacBio: PacBio sequencing platform
CCS: circular consensus sequences
*Msg*: major surface glycoprotein
NGS: next generation sequencing
TGS: third generation sequencing
PCR: polymerase chain reaction
