# Supplementary material for: Distinguishing highly similar gene isoforms with a clustering-based bioinformatics analysis of PacBio single-molecule long reads
Source: BioData Min. 2016 Apr 5;9:13. doi: 10.1186/s13040-016-0090-8 (PMC4820869; doi:10.1186/s13040-016-0090-8)
Supplement: Additional file 1: Table S1. — Representative results of failure of de novo assembly of 454 reads for an ~1.5 kb segment of 10 known msg isoforms of P. jirovecii. (DOCX 18 kb) [file 13040_2016_90_MOESM1_ESM.docx]

**Additional file 1: Table S1.** Representative results of the failure of *de novo* assembly of 454 reads for an ~1.5 kb segment of 10 known *msg* isoforms of *P. jirovecii*.

| Selected assembly programs | Examples of key parameters | No. of contigs  (0.5 - 1.5 kb) | No. of contigs  ( >1 kb) | No. of contigs matching one of the 10 expected *msg* isoforms (with > 99% similarity and ~ 1.5 kb length |
| --- | --- | --- | --- | --- |
| 454 Newbler | seed step =12; seed length =16;  Min. overlap percentage =95% | 14 | 4 | 2 |
| CLC Bio | word size=20; bubble size=30; Similarity=95% | 1 | 4 | 1 |
| MIRA | Pre-defined “454_SETTINGS” | 124 | 31 | 1 |
| Vicuna | word size=20; min consensus base ratio=97% | 1 | 0 | 0 |
| Trinity | K-mer size=25; min. percent similarity for merging = 97% | 9 | 5 | 0 |
