# Supplementary material for: Distinguishing highly similar gene isoforms with a clustering-based bioinformatics analysis of PacBio single-molecule long reads
Source: BioData Min. 2016 Apr 5;9:13. doi: 10.1186/s13040-016-0090-8 (PMC4820869; doi:10.1186/s13040-016-0090-8)
Supplement: Additional file 2: Table S2. — Explanation of the Uclust-based analysis pipeline in details. (DOC 27 kb) [file 13040_2016_90_MOESM2_ESM.doc]

Additional File 2 Table S2: Explanation of the Uclust-based analytic pipeline in details. Exampled input and output are listed.

| **Step** | **Program** | **key Parameters** | **Input Data and Format** | **Output Data and Format** | **Note** | **Exampled Data** |
| --- | --- | --- | --- | --- | --- | --- |
| **Step 1. Unsupervised Clustering** | Uclust | --identity 0.90,  --minlen 600 and other parameters in default settings | PacBio CCS long reads (typically 5000 - 10,000) in FASTA format | Each read is assigned with a cluster groups in Uclust cluster format | Shorter reads and singleton reads are discarded | Addl_file3: step1_input-pacbio-ccs-read.fasta |
| **Step 2. Building a consensus sequence (repeat this step for each cluster from output of step1)** | MUSCLE | default settings | The output from step1: Multiple reads belonging to the same Uclust group in FASTA format | Reads in aligned FASTA format; A Consensus sequence in aligned FASTA format | Run this step interactively for each of read cluster; Double stranded consensus sequences are collected as extra positive evidence for next step. | Addl_file3: Step1_output_Step2_input-uclust-cluster.uc |
| **Step 3. Cleaning up redundancy** | Sequencher | --identity 0.93,  --overlap 100 bp and others in default settings | Collection of output from step 2: Total consensus sequences in FASTA format | Cleaned and final contig sequences in FASTA format | Contigs only with single stranded consensus sequences are filtered | Addl_file3:  Step2_output_Step3_input-consense-sequence.fasta Step3_output-final-contig.fasta |
